# Data on the Relationships of Signal-To-Cutoff Ratios of Elecsys HIV Antigen/Antibody and Elecsys Syphilis Assays to Subsequent Confirmatory Testing at an Academic Medical Center

**DOI:** 10.1016/j.dib.2021.107549

**Published:** 2021-11-06

**Authors:** Saurav Chopra, Matthew D. Krasowski

**Affiliations:** Department of Pathology, University of Iowa Hospitals and Clinics, 200 Hawkins Drive, Iowa City, IA 52242, USA

**Keywords:** False positive reaction, HIV-1, immunoassay, syphilis, *Treponema pallidum*, viral load

## Abstract

Immunoassays are widely used as screening tests for HIV and syphilis in clinical, public health, and research settings. For syphilis, immunoassays are used in 'reverse syphilis algorithms' that start with treponemal tests such as syphilis IgG or syphilis total antibodies for the initial screen. Many screening immunoassays for HIV and syphilis use signal-to-cutoff (S/CO) values for determining positivity, with a cutoff value such as 1.0 differentiating positive from negative. Published literature indicates that the S/CO value often correlates with the likelihood of subsequent confirmation of HIV and syphilis infections, with low S/CO values barely exceeding the cutoff more likely to represent false positive screens. The data in this article present results from the Roche Diagnostics Elecsys HIV combi PT Assay and the Elecsys Syphilis Assay. The data include 19,368 syphilis total antibody screening results on 15,764 unique patients and 28,952 HIV screening results on 24,556 unique patients, S/CO values, clinical area where testing was ordered, sex, and age. For samples with positive syphilis total antibody screens, the data also include results of RPR (the immediate next step in the reverse algorithm), *Treponema pallidum* particle agglutination (TP-PA; for those samples in which RPR was non-reactive in the testing cascade), and clinical information and other testing related to diagnosis of syphilis. For positive HIV screens, the data also include HIV antibody differentiation results, HIV-1 PCR or HIV-2 results results (if performed), and clinical information related to diagnosis of HIV. The distributions of S/CO values relative to confirmation status were analyzed.

## Specifications Table

**Table:** 

Subject	Medicine and Dentistry
Specific subject area	Pathology and Medical Technology
Type of data	Figs. Table Supplemental files
How data were acquired	Retrospective chart and data review from laboratory analysis performed at an academic medical center central clinical laboratory were obtained via tools within the electronic medical record.
Data format	Raw and Analyzed
Parameters for data were collection	Retrospective data on all syphilis screening and confirmatory tests were obtained from the electronic medical record (Epic, Inc.) covering the time period from April 2, 2018 through May 14, 2021. Retrospective data on all HIV screening and confirmatory tests was obtained from the electronic medical record covering the time period from July 17, 2018 through May 14, 2021. Detailed chart review was performed for all cases with a reactive syphilis antibodies result or a positive HIV screen. The project had approval from the University of Iowa Institutional Review Board.
Description of data collection	There were a total of 19,368 syphilis total antibody tests performed on 15,764 unique patients during the retrospective analysis period. There were a total of 28,952 HIV screening tests performed on 24,556 unique patients during the retrospective analysis period. For patients with reactive syphilis antibodies, the data collection included results of subsequent testing performed as part of the reverse syphilis algorithm, namely rapid plasma reagin (RPR) and *Treponema pallidum* particle agglutination (TP-PA), as well as clinical follow-up and information related to syphilis diagnosis for patients with samples that had positive syphilis total antibody tests. For patients with positive HIV screens, the data collection also contain results of confirmatory HIV testing (antibody differentiation and/or HIV PCR) and clinical follow-up and information related to HIV diagnosis for patients with positive HIV screens. Accounting for patients who had both HIV and syphilis testing performed, there were a total of 27,502 unique patients combining both datasets. The de-identified code numbers for individual patients do not correspond across the HIV and syphilis datasets.
Data source location	University of Iowa Hospitals and Clinics, Iowa City, Iowa, United States of America
Data accessibility	Two tables and four figures are included within the paper. 4 Supplementary files are deposited in two different Mendeley datasets for the syphilis and HIV syphilis data: **Syphilis** Data identification number: 10.17632/9n68d47rbr.3 Direct URL to data: https://data.mendeley.com/datasets/9n68d47rbr/3 **HIV** Data identification number: 10.17632/jvwtc23k46.4 Direct URL to data: https://data.mendeley.com/datasets/jvwtc23k46/4

## Value of the Data

• The data provided are of value as HIV and syphilis testing is widely performed for clinical, public health, and research purposes.
• Clinicians, other researchers, or personnel in clinical laboratories might find this data useful as a reference for comparison.
• Our data set would serve as a starting point for researchers interested in future investigations investigating the reverse syphilis algorithm.
• The data provide information on false positive HIV and syphilis testing in a population with overall low prevalence for HIV and syphilis.
• The data include 19,368 syphilis total antibody screening results on 15,764 unique patients and 28,952 HIV screening results on 24,556 unique patients.

## 1 Data Description

**Fig. 1:**
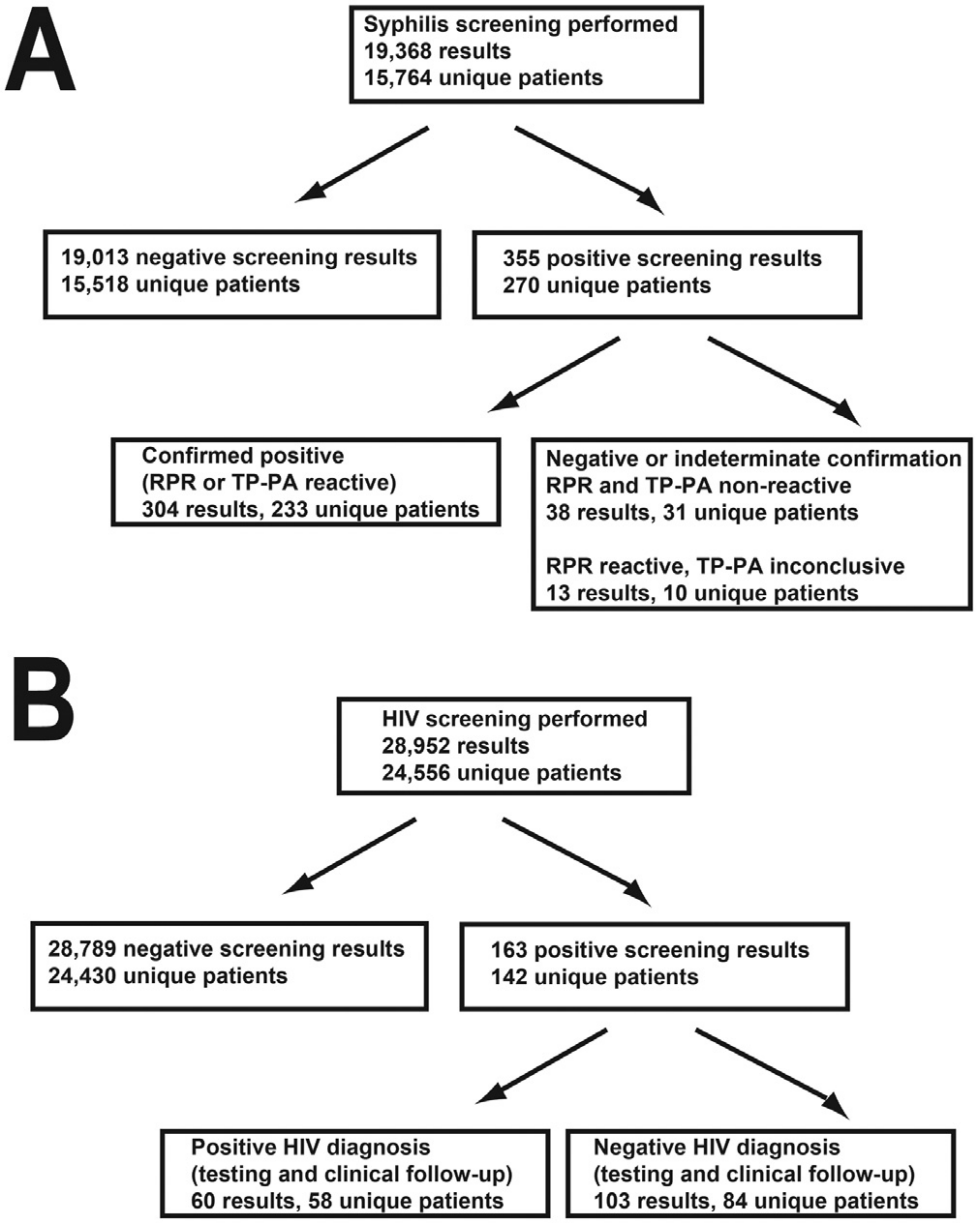
Flow chart of (A) syphilis and and (B) HIV testing for the retrospective study. Some patients fit into more than one category which affects unique patient totals in each subgroup. Abbreviations: RPR, rapid plasma reagin; TP-PA, *Treponema pallidum* particle agglutination.

For syphilis testing, the retrospective analysis in the present study includes detailed data on 19,368 samples from 15,764 unique patients who had syphilis testing using the reverse algorithm performed at an academic medical center. Fig. 1 (panel A) shows a flow diagram of the syphilis testing in the retrospective study. Multiple studies have shown an association of the signal-to-cutoff (S/CO) value of syphilis immunoassay tests with subsequent positive confirmatory testing indicating either past or current infection [1], [2], [3], [4]. In the reverse algorithm, confirmation testing typically entails RPR as the next test following a positive syphilis antibodies result [5]. If RPR is non-reactive, then another treponemal test such as TP-PA or fluorescent treponemal antibody absorption (FTA-ABS) is performed. Positive syphilis antibodies in conjunction with reactive RPR are common in active or resolving syphilis infection. Positive syphilis antibodies with non-reactive RPR but a reactive TP-PA or FTA-ABS most often indicates prior treated syphilis or late/latent syphilis. If the RPR and the second treponemal test (TP-PA or FTA-ABS) are both non-reactive, this often represents a “false positive” for actual past or present syphilis infection [6].

For HIV testing, the retrospective analysis in the present study includes detailed data on 28,952 samples originating from 24,556 unique patients who had HIV screening tests ordered at an academic medical center. Fig. 1 (panel B) shows a flow diagram of the HIV testing in the retrospective study. Similar to syphilis antibodies, multiple studies have shown an association of the S/CO value of HIV screening tests with subsequent verification of HIV diagnosis using confirmatory testing and clinical information [7], [8], [9], [10], [11], [12], [13], [14], [15]. Confirmation testing commonly used in the United States and some other countries includes antibody differentiation assays (in the present study was the Bio-Rad Geenius assay) and HIV RNA PCR. For the present study, a true (confirmed) positive required positive HIV RNA PCR and clinical documentation of HIV diagnosis. A non-true (false) positive would be indicated by a positive HIV screen with negative confirmatory testing (especially HIV PCR) and lack of a clinical diagnosis of HIV. We did extensive chart review on all cases with a positive HIV screen.

**Fig. 2:**
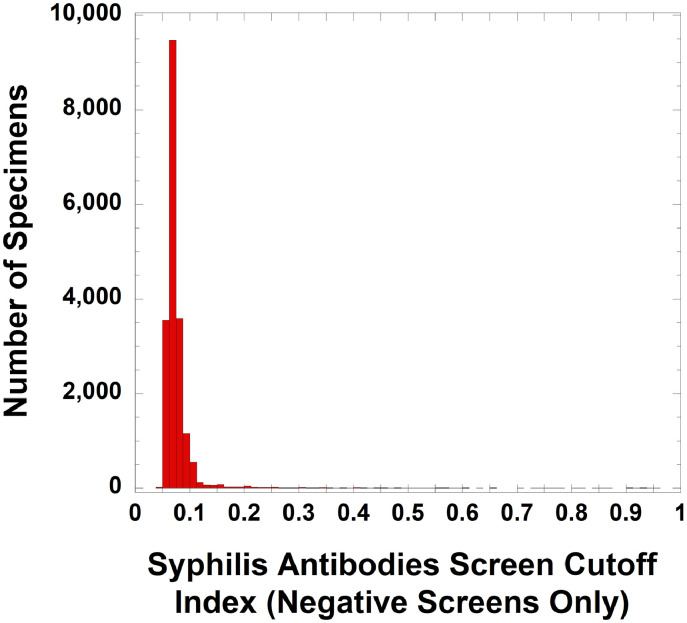
Distribution of S/CO ratios of negative (non-reactive) Roche Diagnostics Elecsys Syphilis assay screens. The data includes 19,013 negative syphilis antibody tests on 15,517 unique patients.

**Fig. 3:**
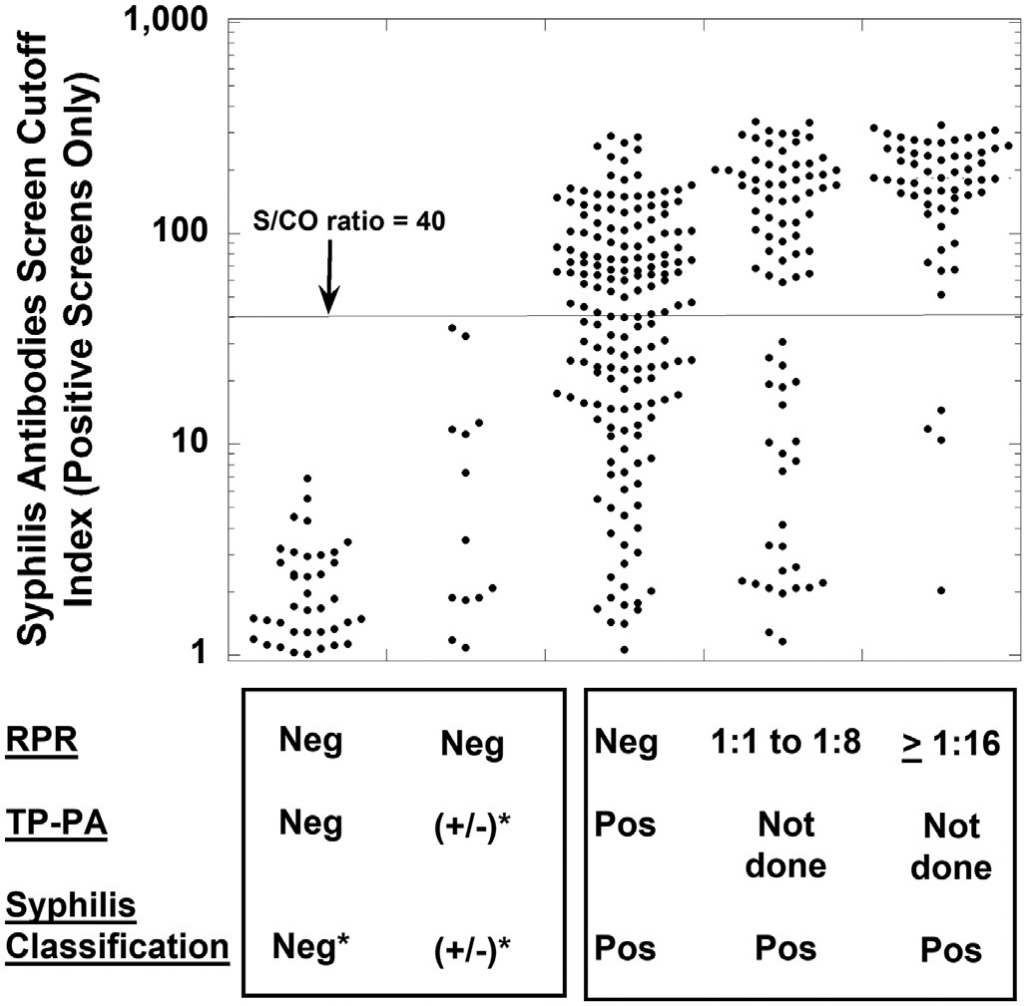
Distribution of S/CO ratios for five categories of samples with a positive syphilis antibodies screen (from left to right, reactive indicated as “Pos” and non-reactive as “Neg”): RPR and TP-PA both non-reactive (38 results on 31 unique patients), RPR non-reactive and TP-PA inconclusive (indicated as “+/-”; 13 results on 10 unique patients), RPR non-reactive and TP-PA reactive (167 results on 130 unique patients), RPR reactive with a titer of 1:1 to 1:8 (79 results on 66 unique patients, and RPR reactive with a titer of 1:16 or higher (58 results on 57 unique patients). The first two categories are either considered negative or inconclusive (“+/-”) for past or present syphilis infection. The latter three categories are considered confirmed positive results, although can occur with active, latent, or past treated syphilis infections. Note that samples tested from some patients fell into more than one category categories (e.g., an RPR titer of 1:16 or higher result in one specimen and a different result of RPR non-reactive/TP-PA reactive in another specimen). There were a total of 270 unique patients for the 355 samples with a positive syphilis screen (233 patients confirmed positive by either a reactive RPR or reactive TP-PA, 31 patients had an overall false positive interpretation due to non-reactive RPR and TP-PA results, and 6 patients who only had inconclusive TP-PA results after a positive syphilis screen in the retrospective timeframe). No false positives were seen with an S/CO ratio exceeding 40. However, results consistent with past or current syphilis infection spanned a wide range of S/CO values.

For syphilis testing, Table 1 shows demographics of the population being tested for syphilis by the reverse algorithm, including the clinical locations and specialties associated with orders.Fig. 2 shows the distribution of S/CO values for all negative syphilis screens (defined according to the package insert instructions as an S/CO value less than 1.00), encompassing 19,013 negative syphilis screening tests on 15,518 unique patients. Fig. 3 is a dot plot of S/CO values for five groups that initially had a positive syphilis antibodies result (total of 355 results on 270 unique patients): (A) RPR and TP-PA both non-reactive (38 results on 31 unique patients), (B) RPR non-reactive and TP-PA inconclusive (13 results on 10 unique patients), (C) RPR non-reactive and TP-PA reactive (167 results on 130 unique patients), (D) RPR reactive with a titer of 1:1 to 1:8 (79 results on 66 unique patients), and (E) RPR reactive with a titer of 1:16 or higher (58 results on 57 unique patients). Categories (C) through (E) would be considered positive by confirmation for syphilis, representing either past or present syphilis infection. For categories (D) and (E), TP-PA would not be performed, because the RPR is reactive and no further testing is needed based on the reverse algorithm. The raw data for the syphilis study are included in Supplementary files 1 and 2. In Table 1 and Fig. 3, there are patients with multiple test results that fell into two or more categories. This is explained in more detail in the footnote to Table 1 and the legend for Fig. 3. In the retrospective timeframe, there were only 24 unique patients who had negative syphilis screening results but then screened positive for syphilis at a later timepoint.

**Table 1:** Demographics of syphilis testing.

	Summary data
**Location where syphilis screening was ordered (based on 19,368 syphilis tests on 15,764 unique patients)**	
Emergency Department	315 (1.6%)
Infectious Disease Clinic	1368 (7.1%)
Inpatient	977 (5.0%)
Obstetrics	5101 (26.3%)
Outpatient (other than infectious disease or obstetrics)	11,607 (59.9%)
**Specialties ordering syphilis screening the most**	
Obstetrics-gynecology	5101 (26.3%)
Family medicine	2582 (13.3%)
Transplant services	1823 (9.4%)
Infectious diseases	1368 (7.1%)
Internal medicine	1208 (6.2%)
All other specialties	7286 (37.6%)
**Confirmed positive (RPR or TP-PA reactive; 304 results on 233 unique patients)**^1^	
Mean age (yrs) ± SD	40.8 ± 14.4
Median age (yrs)	37.5
Female / male (unique patients)	60 / 173
**False positive screen (initial screen positive, RPR and TP-PA both non-reactive; 38 results on 31 unique patients)**^1^	
Mean age (yrs) ± SD	39.3 ± 15.6
Median age (yrs)	36.0
Female: male (unique patients)	13 / 18

1 There were 10 unique patients (4 females, 6 males; 13 total results) who had one or more RPR non-reactive and TP-PA inconclusive results after a positive syphilis total antibodies screen. Three of these patients later had a reactive TP-PA result and are included in unique patient total for the “Confirmed Positive” category above. One patient later had a non-reactive TP-PA and is included in the unique patient total for “False Positive Screen” category. The remaining 6 patients only had inconclusive TP-PA results in the retrospective timeframe and are not included in either Confirmed Positive or False Positive Screen category due to indeterminate conclusion. Thus, there were a total of 270 unique patients (233 confirmed positive, 31 false positive, and 6 who only had inconclusive TP-PA results after a positive screen). There were no example of patients who had both a false positive and confirmed positive screen in the retrospective timeframe.

**Fig. 4:**
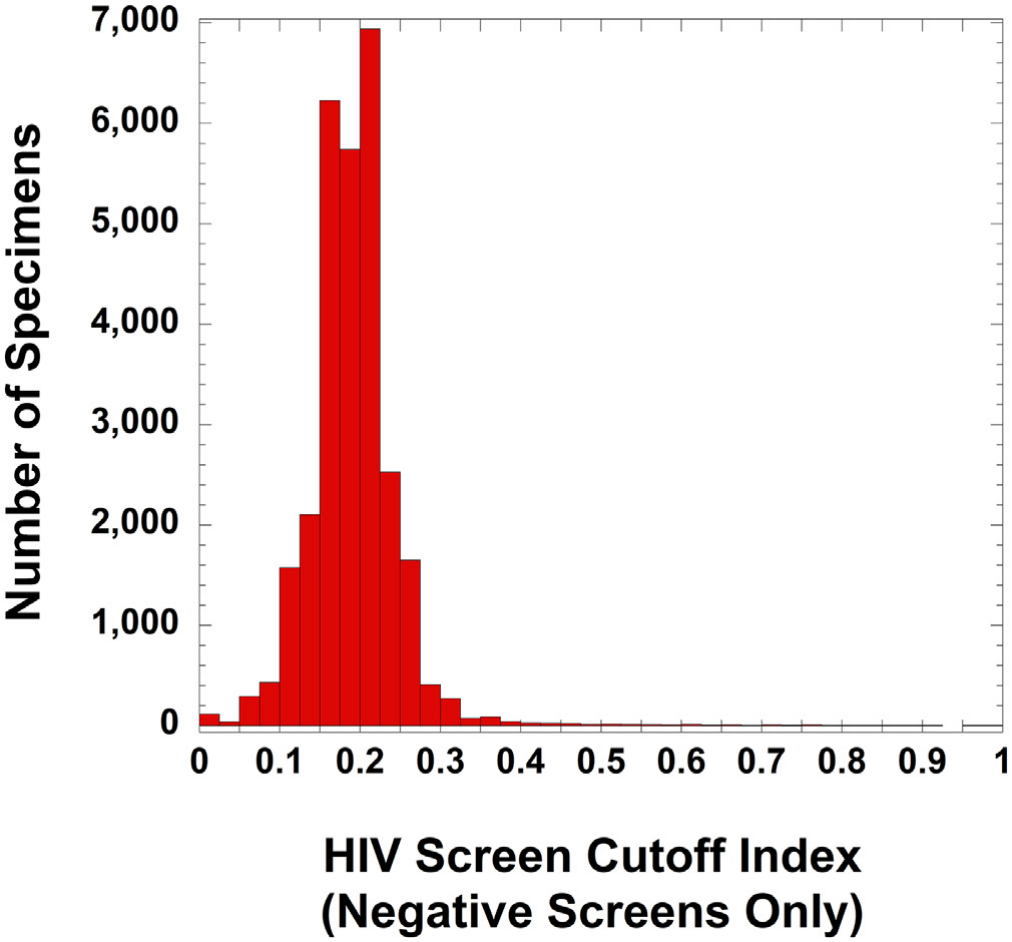
Distribution of S/CO ratios of negative (non-reactive) Roche Diagnostics Elecsys HIV combi PT screens. The data includes 28,789 negative HIV screening tests on 24,430 unique patients.

**Fig. 5:**
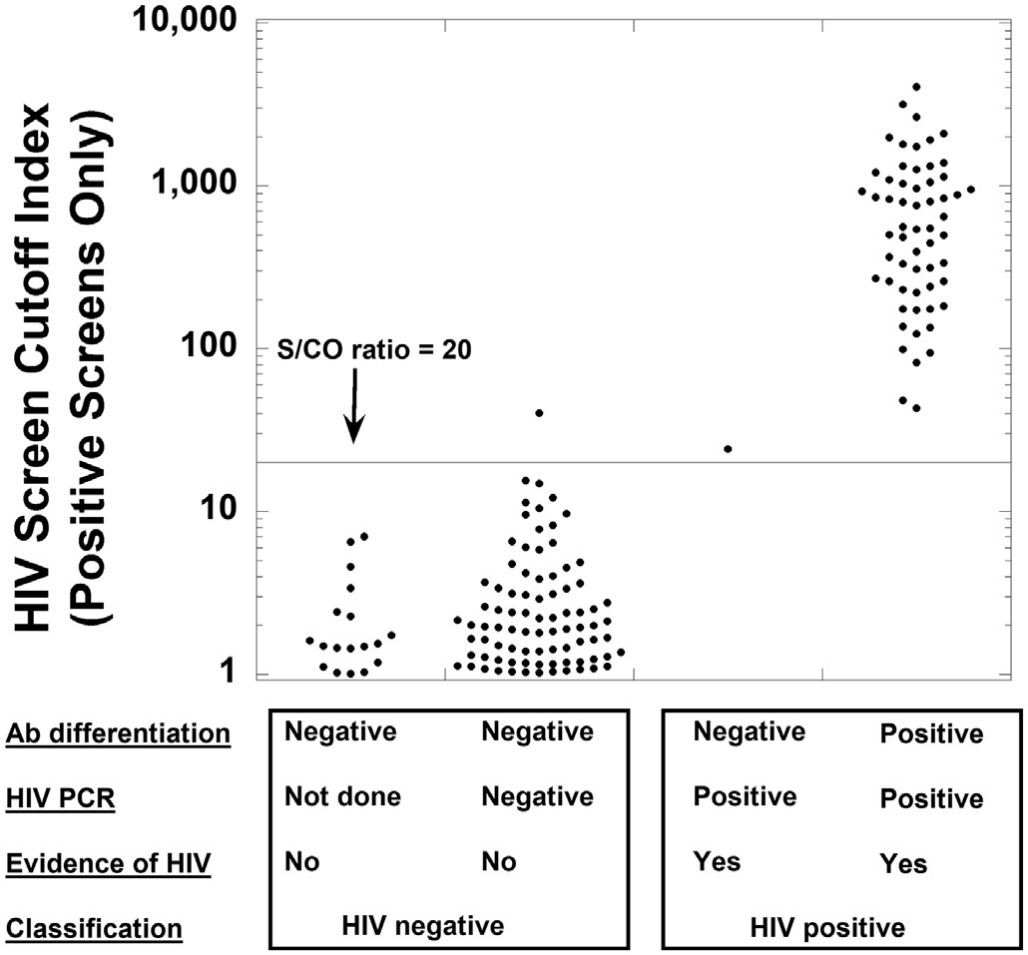
Distribution of S/CO ratios for four categories of samples with a positive (reactive) HIV screen (from left to right): samples with negative antibody differentiation, no subsequent HIV PCR, but with follow-up clinical data showing no evidence of HIV infection (18 samples from 17 unique patients); samples with negative antibody differentiation, one or more subsequent negative HIV PCR results, and no follow-up evidence of HIV infection (85 samples on 67 unique patients); negative antibody differentiation but positive HIV PCR and clinical evidence of HIV (1 sample on 1 patient); and samples with positive antibody differentiation, positive HIV PCR, and clinical evidence of HIV (59 samples from 57 unique patients). The first two categories are collectively grouped as “HIV negative” status and comprise 103 results on 84 unique patients. The latter two categories are collectively grouped as “HIV positive” status and comprise 60 results on 58 unique patients. An S/CO cutoff of 20 captures all confirmed positive cases and excludes all but one of the false positives.

For HIV testing, Table 2 shows demographics of the population being tested for HIV, including the clinical locations and specialties associated with orders. Fig. 4 shows the distribution of S/CO values for all negative HIV screens (defined according to the package insert instructions as an S/CO value less than 1.00), encompassing 28,789 negative HIV screening tests on 24,430 unique patients. Fig. 5 is a dot plot of S/CO values for four groups with a positive HIV screen (total of 163 samples on 142 unique patients): (A) positive HIV screen with negative antibody differentiation, clinical follow-up demonstrating lack of actual HIV infection, but without documentation of follow-up HIV PCR testing (18 samples from 17 unique patients); (B) positive HIV screen, negative antibody differentiation (Geenius assay), negative HIV PCR testing, and clinical follow-up demonstrating lack of actual HIV infection (85 samples on 67 unique patients); (C) positive HIV screen, negative antibody differentiation (Geenius assay), positive HIV PCR testing, and documented clinical HIV diagnosis (only one patient and one sample was in this category); and (D) positive HIV screen, positive antibody differentiation, positive HIV PCR testing, and documented clinical HIV diagnosis (59 samples on 57 unique patients). In the retrospective timeframe, there were 16 unique patients who had negative HIV screening results but then had a positive HIV screen at a later timepoint. Of those 16 patients, only 2 ultimately were assigned a clinical diagnosis of HIV by subsequent testing and follow-up. There were no examples of patients who had a false positive HIV screen and then later had clinical diagnosis of HIV.

**Table 2:** Demographics of HIV testing.

	Summary
**Location where HIV screening was ordered (based on 28,952 HIV screening tests on 24,556 unique patients)**	
Emergency Department	786 (2.7%)
Inpatient	2960 (10.2%)
Outpatient	25,206 (87.1%)
**Specialties ordering HIV screening the most**	
Obstetrics-gynecology	5673 (19.6%)
Family medicine	4745 (16.4%)
Internal medicine	3366 (11.6%)
Transplant services	1804 (6.2%)
All other specialties	13,364 (46.2%)
**Confirmed positive screen by HIV PCR and clinical history (60 results on 58 unique patients)**	
Mean age (yrs) ± SD	38.2 ± 15.7
Median age (yrs)	37.5
Female / male	22 / 36
**False positive screen (initial screen positive, not confirmed by HIV PCR or clinical history; 103 results on 84 unique patients)**	
Mean age (yrs) ± SD	37.7 ± 16.0
Median age (yrs)	35.0
Female: male	45 / 39

The raw data for the HIV study are included in Supplementary files 3 and 4.

• Supplementary file 1: Data for 19,368 syphilis screening tests on 15,764 unique patients using the Roche Diagnostics Elecsys Syphilis assay. All laboratory data involve analysis on plasma or serum. Specific data fields include: unique patient identification number (deidentified), whether result was first or only syphilis screening test performed for the specific patient within the retrospective timeframe or a repeat test (with days after initial test recorded), age in years, sex (as recorded in the electronic medical record), clinical location/unit at time of testing (emergency department, infectious disease clinic, obstetrics, inpatient, or outpatient clinic other than infectious diseases or obstetrics), syphilis screening result (negative or positive), and S/CO value for the syphilis screening.
• Supplementary file 2: Data for 355 positive syphilis screening tests on 270 unique patients using the Roche Diagnostics Elecsys Syphilis assay. All laboratory data involve analysis on plasma or serum. Specific data fields include: unique patient identification number (deidentified), whether result was first or only syphilis screening test performed for the specific patient within the retrospective timeframe or a repeat test, age in years, sex (as recorded in the electronic medical record), clinical location/unit at the time of syphilis testing (emergency department, infectious disease clinic, obstetrics, inpatient, or outpatient), S/CO value, results of subsequent testing in the reverse algorithm cascade (classified into the following categories: RPR reactive with specific titer noted, RPR and TP-PA non-reactive, RPR non-reactive / TP-PA inconclusive, and RPR non-reactive / TP-PA reactive), specific category (including RPR reactive results broken down into 1:1 to 1:8 and 1:16 or higher RPR titer subgroups), and clinical notes.
• Supplementary file 3: Data for 28,952 HIV screening tests on 24,556 unique patients using the Roche Diagnostics Elecsys HIV combi PT assay. All laboratory data involve analysis on serum or plasma. Specific data fields include: unique patient identification number (deidentified), whether result was first or only HIV screening test performed for the specific patient within the retrospective timeframe or a repeat test (further indicating days after initial testing), age in years, sex (as recorded in the electronic medical record), clinical location/unit at time of testing (emergency department, infectious disease clinic, obstetrics, inpatient, or outpatient), HIV screening result (negative or positive), and S/CO value.
• Supplementary file 4: Data for 163 positive HIV screening tests on 142 unique patients using the Roche Diagnostics Elecsys HIV combi PT assay. All laboratory data involve analysis on serum or plasma. Specific data fields include: unique patient identification number (deidentified), whether result was first or only HIV screening test performed for the specific patient within the retrospective timeframe or a repeat test (further indicating days after initial testing), age in years, sex (as recorded in the electronic medical record), clinical location/unit at time of testing (emergency department, infectious disease clinic, obstetrics, inpatient, or outpatient), S/CO value, result of antibody differentiation assay (Bio-Rad Geenius antibody differentiation), results of HIV RNA PCR, days between HIV RNA PCR and the antecedent HIV screen, HIV results classification (Confirmed Positive, Confirmed Positive but Geenius Negative), False Positive/PCR negative, False Positive/PCR not done), and clinical notes.

## 2 Experimental Design, Materials and Methods

All data was obtained from patient data in the electronic medical record from the University of Iowa Hospitals and Clinics (Iowa City, Iowa, United States). A reporting tool within the electronic medical record, known as Epic Reporting Workbench [16], was used to identify all syphilis and HIV screening tests performed in the retrospective timeframes. Only data from patients who had syphilis or HIV testing performed at the University of Iowa Hospitals and Clinics were included; no data was obtained from diagnostic vendors of any of the laboratory assays used for clinical testing. During the retrospective analysis period for syphilis testing (April 2, 2018 through May 14, 2021), syphilis screening using the reverse algorithm was performed using the Roche Diagnostics Elecsys Syphilis assay (Roche Diagnostics, Indianapolis, IN). During the retrospective analysis period for HIV testing (July 17, 2018 through May 14, 2021), HIV screening was performed using the Roche Diagnostics Elecsys HIV combi PT (Roche Diagnostics, Indianapolis, IN). In a previous study, we reported the relationship of S/CO ratio to confirmation for two other HIV assays (Abbott Architect HIV Ag/Ab Combo Assay, a 4th generation HIV combination assay; Bio-Rad Bioplex 2200 HIV Ag-Ab Assay, a 5^th^ generation HIV assay) [12].

The Elecsys Syphilis assay is an immunoassay for the detection of the total antibodies (especially IgG and IgM) to *Treponema pallidum* in the human plasma and serum and outputs a single S/CO ratio that translates into a positive or negative qualitative result [17]. An S/CO ratio of 1.00 or greater on the Elecsys assay is considered positive, with repeat testing to verify the result. For samples with a positive syphilis antibodies screen, subsequent confirmatory testing includes first an RPR (Bio-Rad Laboratories Bioplex 220 Syphilis Total & RPR Assay, Redmond, WA). If RPR is reactive, no further testing is done. If RPR is non-reactive, then TP-PA testing (referred to reference laboratory, ARUP Laboratories, Salt Lake City, UT) is then done.

The Elecsys HIV combi PT is a 4^th^ generation HIV assay that simultaneously detects HIV-1 p24 antigen along with antibodies to HIV-1 and HIV-2 and outputs a single signal-to-cutoff (S/CO) ratio that translates into a reactive/nonreactive (referred to as positive/negative in this manuscript) qualitative result [13,^18^,19]. An S/CO ratio of 1.00 or greater on the Elecsys assay is considered positive, with repeat testing to verify the result. For all samples with a positive HIV screen, antibody differentiation was performed with the Bio-Rad Geenius HIV-1/HIV-2 Antibody Differentiation assay (Bio-Rad Laboratories, Redmond, WA), an assay that can confirm HIV infection. Final confirmation of HIV infection utilized HIV RNA PCR, testing which required a new specimen from the patient.

## Ethics Statement

The analyses had approval by the University of Iowa Institutional Review Board (protocols # 202001165 and 202105258) as a retrospective project.

## CRediT Author Statement

**Saurav Chopra:** Formal analysis, Writing – Review & Editing, Visualization. **Matthew D. Krasowski:** Formal analysis, Conceptualization, Writing – Original Draft, Writing – Review & Editing, Methodology, Supervision.

## Declaration of Competing Interest

The authors declare that they have no known competing financial interests or personal relationships that could have appeared to influence the work reported in this paper.
